# Acute Nephritis with Extreme Hæmaturia

**Published:** 1908-03-21

**Authors:** 


### Medicine.

### ACUTE NEPHRITIS WITH EXTREME HÆMATURIA.

The case, which was recently described in this journal, of acute nephritis with extreme hæmaturia, is, as we said at the time, unusual; but to show that it is not extremely uncommon we give the following four cases, in which hæmaturia was so profuse that at first it looked as if there must be something besides acute Bright's disease to account for it. Renal calculus or villous tumour seemed possible, but the course of the illness indicated that the hæmaturia was due solely to the nephritis.

The first two cases further illustrate the fact that sepsis is a possible cause of acute Bright's disease, presumably as the result of absorption of cocci or their toxins. The third case shows that scarlet fever may be so slight as to escape detection, and yet be followed by extremely acute nephritis. The fourth case suggests that nephritis may follow scarlet fever without giving rise to obvious symptoms at the time, and yet leave the kidneys in a condition ripe for renewed inflammation on the slightest provocation.

The first case is that of a young man, aged twenty-three, who had not long since suffered from acute gonorrhœa, with suppurating buboes in the groins. The patient had been careless in following the treatment advised, and shortly developed acute pains in many joints. During his illness he also had well-marked pericarditis; but it seemed less likely that the joint troubles and pericarditis were rheumatic than that they were either gonococcal, or even pyæmic, due to absorption of staphylococci from the local sepsis in connection with the penis and buboes. He ultimately got quite well, without any valvular heart disease, but also without permanent stiffness of any joint such as might be expected from gonococcal or staphylococcal arthritis. The joints, however, were affected for several months before they finally got well.

Almost from the beginning of the joint pains, this man had passed blood in his urine, and the degree of hæmaturia was at times extreme. There was no pus present beyond what could be accounted for by the urethral condition. There were no crystals. There were, on the other hand, large numbers of renal epithelial cells, and tube casts of various kinds, though these were difficult of detection while the hæmaturia was at its height. The urine went almost solid with coagulated albumen on boiling, and there seemed no doubt that there was a very acute non-suppurative nephritis. The patient complained of a dull sense of weight in both loins, which pointed to this conclusion, although there was but a slight degree of œdema of the ankles and eyelids in the case. It is very likely that the main brunt of the inflammation here fell upon the glomeruli, and that had there been any opportunity of examining the kidneys they would have been of the large red blood-dripping variety. The degree of hæmaturia in such cases doubtless depends on irritation of the glomeruli by the poison which is being eliminated, with consequent afflux of much blood to them; if the engorgement attains a certain intensity, the delicate vessels of the malpighian bodies give way, and much blood escapes directly into the tubules.

Hæmaturia may be caused by the irritation of the kidneys excited by a substance which can reach these glands only through the blood. Both turpentine and cantharides will irritate the kidneys when administered in large doses. Cantharides in small doses excites the kidneys and increases the secretion of urine; but large doses diminish the flow of urine, the state of engorgement being too great to be compatible with the healthy functions of the organ; finally, the vessels yield, and hæmorrhage is the result. The effect of turpentine is very similar. Oxalic acid has a like action, upon whi h little stress is laid as a rule, though in cases of attempted suicide by its use, acute nephritis with hæmaturia is not at all uncommon during recovery. There is no need to detail the course of the illness in this patient. The hæmaturia continued severe for a fortnight, and then got less as the joint pains and the pericarditis lessened, and finally disappeared entirely.

The second case is that of a labourer, aged forty. He had been temperate in his habits, but for some time before his admission to hospital he had been getting into a bad state of health, and had had subcutaneous abscesses in his axilla and elsewhere during the preceding fortnight. Soon after these appeared, œdema came on, first in his feet, but very soon afterwards in his upper extremities and face; finally, effusion took place into his scrotum and abdomen; and when he entered the hospital he was universally dropsical. The urine was very scanty, amounting to no more than four ounces in twenty-four hours. It had a specific gravity of 1020, and it was like pure blood in appearance. It coagulated solid on boiling. Microscopically, in addition to myriads of red corpuscles, a great number of various kinds of renal tube casts were found, together with an abundance of renal epithelial cells. There were no particles of new growth, nor any crystals. The extreme hæmaturia seemed due entirely to acute Bright's disease, and this in turn could only be attributed to absorption of staphylococci or their toxins from the septic foci in the axilla and elsewhere. There had been no scarlet fever in the man himself or in any with whom he had been in contact, so far as could be ascertained.

The usual line of treatment for acute nephritis was adopted, including hot-air baths, and cupping of the loins, on account of the suppression of urine that threatened. This was upon May 14. By May 30 the secretion of urine was fairly re-established, and the degree of hæmaturia had simultaneously lessened. On May 31 two pints and eighteen ounces of urine were passed; on June 1 three pints; after this the blood began to appear in the urine in increasing quantities again, and on June 6 the quantity of urine passed fell below thirty ounces; on the 8th it was twenty-eight ounces. The hæmaturia now returned with greater violence than ever, so much so that the urine seemed almost like pure blood; it also contained an increased number of renal tube casts and kidney epithelial cells. The patient was now extremely anæmic, with pallid lips and gums, hæmic murmurs, and greatly impaired nutritive powers. On pricking the ear, the blood that came was thin and watery. The anæmia was partly due, no doubt, to the former abscesses and general ill-health; but it was being rapidly accentuated by the actual blood loss from the kidneys. It seemed unwise to continue with milk as the chief article of diet, so notwithstanding the existence of the nephritis, plenty of nutritious food and ferruginous wine was ordered, and gallic acid was given in four grain doses four times a day. Improvement slowly but surely ensued. For weeks the hæmaturia was still a marked feature, but ultimately, three months from the beginning, the patient was able to leave the hospital without œdema, without hæmaturia, and with only a trace of albumen in his urine. His anæmia was not completely cured upon his discharge, but it was very greatly alleviated.

The third case was that of a boy, aged four years. He came under observation for general œdema and for severe hæmaturia, with scanty urine. A month previously his brother and sister were taken with scarlet fever, but the patient himself, though he had been constantly with the others, did not seem to take it. He had had no sore throat, no rash, no pyrexia, and his skin was not desquamating. Three days before admission he was first noticed to have a much larger abdomen than usual, and shortly afterwards his ankles, legs, and face were puffy. On admission fluid could be detected in his abdomen, and the anasarca was general. The hæmaturia, though intense for a while, then rapidly decreased; the quantity of urine passed regained its normal amount, and the acute nephritis ran its usual course, and complete restoration of the urine to its natural state occurred in about a month.

It seems very probable that this patient really had scarlet fever at the same time as his brother and sister, but in so mild a degree that it entirely escaped notice. The case exemplifies the fact, not only that acute nephritis may cause extreme hæmaturia, but also that even the slightest attacks of scarlet fever may be followed by nephritis. Indeed, owing to the absence of caution as to exposure to draughts and to damp, the extremely slight cases of scarlatina are perhaps more liable to nephritis than are the others. It opens up the question of whether or not a good many of the apparently causeless cases of acute nephritis in children and young adults may not really be scarlatinal.

The fourth case is that of a girl aged sixteen, who had never menstruated. Two months previous to her admission to the hospital blood had begun to appear in the urine without any apparent cause, and the patient had experienced pains in her limbs, supposed to be rheumatic, shifting from one part to another. At first it was thought that the blood was due to the onset of menstruation, but this was put out of court by its continuance. As days went on, she continued to pass blood in her urine in increasing quantity, until the urine soon came to be bright red with blood; to those who did not know how much colour can be given to a bulk of urine by a much less quantity of blood, it seemed as if she were passing pure blood. The girl rapidly became blanched. A venous murmur was audible in the neck. There were no symptoms of vesical trouble. The blood was uniformly mixed with the urine, and came away as freely at the beginning as at the end of micturition, nor did the urine contain any clots.

It is noteworthy that there was no œdema at all in this case; the diagnosis was obscure, and although Bright's disease was discussed, it was not diagnosed with any certainty until a post-mortem examination was made. The uninterrupted discharge of the large quantity of blood which this girl had been passing for so long a period, without a symptom referable to the bladder or to any other neighbouring organ, left little doubt that the kidney was the source of the hæmorrhage. Microscopical examination of the urinary deposit was very difficut, owing to the myriads of red corpuscles present obscuring the field. The only things noticed with certainty besides the red corpuscles were numerous crystals of oxalate of lime. Had renal tube casts and epithelial cells been observed, the diagnosis would have been comparatively easy; but the oxalate of lime crystals, associated with extreme hæmaturia, pointed rather to a calculus. Against such a diagnosis, of course, there were three points: First, the absence of renal colic; secondly, persistence of the extreme hæmaturia, unabated by rest in bed; and thirdly, the age of the patient—for renal calculi are rare at sixteen. It was found afterwards that the girl had had scarlet fever two years before; there was no history of any dropsy then; but in view of what was found post-mortem it seems likely that there had been nephritis, without symptoms, after the scarlet fever—a thing which there is a good deal of evidence to show to be commoner than might be thought—and that the hæmaturia was due partly to sclerosis after a former nephritis, partly to an acute nephritis superposed upon the older post-scarlatinal trouble. But at the time the cause of the hæmaturia was a mystery. The only treatment seemed to be rest in bed, with a fairly liberal diet on account of the anæmia, and to administer iron and astringents. Lead, gallic acid, tannin, and perchloride of iron, were all tried, but the hæmaturia showed not the least sign of abating. In view of the apparent benefit of gallic acid in a former case, this was pushed in this one, but without benefit. It was clear that the patient was dying of hæmorrhage, and that something more must be done. The next step suggested was the use of some of the terebinthinate medicines, which are supposed to exercise a styptic influence. The necessity for great caution in the use of these remedies is shown by the sequel of this case. The hæmorrhage resisting all the ordinary remedies, five drops of spirits of turpentine were given three times a day. In two days this was followed by strangury, and a great diminution in the quantity of the urine, but not of the blood in it. This was followed by a low febrile state, in which the patient died. At the autopsy the kidneys were mottled and contracted organs in a mixed condition of chronic fibrosis from former nephritis and of more recent acute inflammation.

